# Association between polyunsaturated fatty acid intake and estradiol levels among U.S. women

**DOI:** 10.3389/fnut.2024.1500705

**Published:** 2024-11-20

**Authors:** Lange Guo, Yukui Nan, Kangni Liang, Lizhong Yao, Jiuzhi Li

**Affiliations:** ^1^Department of Urology, People’s Hospital of Xinjiang Uygur Autonomous Region, Ürümqi, China; ^2^Graduate School, Zhejiang Chinese Medical University, Hangzhou, China

**Keywords:** eicosapentaenoic acid, docosapentaenoic acid, polyunsaturated fatty acids, estradiol, NHANES

## Abstract

**Background:**

Polyunsaturated fatty acids (PUFAs) play a crucial role in maintaining homeostasis in the body. However, research on the relationship between PUFA intake and estradiol levels is limited. This study aims to investigate the association between dietary PUFA intake and estradiol levels in women in the United States.

**Method:**

Data on PUFA intake and estradiol levels were drawn from the 2013–2016 National Health and Nutrition Examination Survey (NHANES) for women aged 20 and older. UFA intake was assessed through 24-h dietary interviews, while serum estradiol levels were measured using isotope dilution liquid chromatography–tandem mass spectrometry (ID-LC–MS/MS). Weighted logistic regression models adjusted for covariates were used to analyze the relationship between PUFA intake and estradiol levels. The inflection point of the non-linear relationship between intake of PUFAs and estradiol levels was determined by threshold effects analysis, and a two-part regression model was developed at the inflection point.

**Result:**

Weighted multivariate linear regressions showed positive associations between eicosapentaenoic acid (EPA) and docosapentaenoic acid (DPA) intake and estradiol levels. Even in the fully adjusted model, EPA intake remained positively associated with estradiol levels in the menopausal (*β* = 78.08, 95% CI: 33.58, 122.58; *p* = 0.0006), non-menopausal (*β* = 287.61, 95% CI: 177.29, 397.94; *p* < 0.0001), and total-participant groups (*β* = 208.38, 95% CI: 139.81, 276.95; *p* < 0.0001), and DPA intake remained positively associated with estradiol levels in the non-menopausal (*β* = 318.87, 95% CI: 28.93, 608.82; *p* = 0.0313) and total-participant groups (*β* = 208.03, 95% CI: 22.89, 393.18; *p* = 0.0277). In the two-part regression model, EPA intake greater than 0.09 (*p* < 0.0001) and DPA intake greater than 0.05 (*p* = 0.0033) were positively associated with estradiol levels in non-menopausal women.

**Conclusion:**

This study suggests that higher intake of EPA and DPA in non-menopausal women is associated with increased estradiol levels. These findings support the importance of dietary components in regulating female reproductive health and hormone levels.

## 1 Introduction

Estradiol is a crucial sex hormone in women, produced by the ovaries, that plays a significant role in reproduction and sexual health (1). Lower estradiol levels negatively impact both sexual and non-sexual aspects of an individual’s quality of life (2). Sexual symptoms may include low libido, erectile dysfunction, and difficulties in achieving orgasm (3, 4). Non-sexual symptoms often include fatigue, poor concentration, depression, reduced muscle mass, weakened bone strength, and impaired iron metabolism (5–7). Given its wide-ranging effects, addressing estradiol deficiency is essential for overall health (8).

Polyunsaturated fatty acids (PUFAs) are fatty acids containing two or more double bonds, with omega-3 and omega-6 as the primary types. Omega-3 fatty acids include *α*-linolenic acid (ALA), stearidonic acid (SDA), eicosapentaenoic acid (EPA), docosapentaenoic acid (DPA), and docosahexaenoic acid (DHA). Omega-6 fatty acids comprise linoleic acid (LA) and arachidonic acid (AA). LA and ALA are essential fatty acids primarily derived from crop seeds, vegetable oils, and cereal products (9, 10). Although EPA and DHA are long-chain omega-3 fatty acids, they can also be synthesized from ALA (11). PUFAs are essential for maintaining homeostasis in the body. An imbalance in the omega-6 to omega-3 ratio is associated with the development of modern diseases, including cardiovascular disease, cancer, inflammation, and autoimmune disorders (12, 13).

Despite the recognized importance of PUFAs, clinical research on their intake and their impact on estradiol levels remains limited. This study aims to investigate the association between PUFA intake and estradiol levels, providing valuable insights for potential dietary interventions aimed at hormone regulation.

## 2 Materials and methods

### 2.1 Data source

This study utilized data from two cycles of the National Health and Nutrition Examination Survey (NHANES), a nationally representative cross-sectional survey conducted biennially by the National Center for Health Statistics (NCHS) on the non-institutionalized U.S. population. NHANES employs a complex, multistage probability sampling design, gathering data through in-person interviews, physical examinations, and laboratory tests.

**Figure 1:**
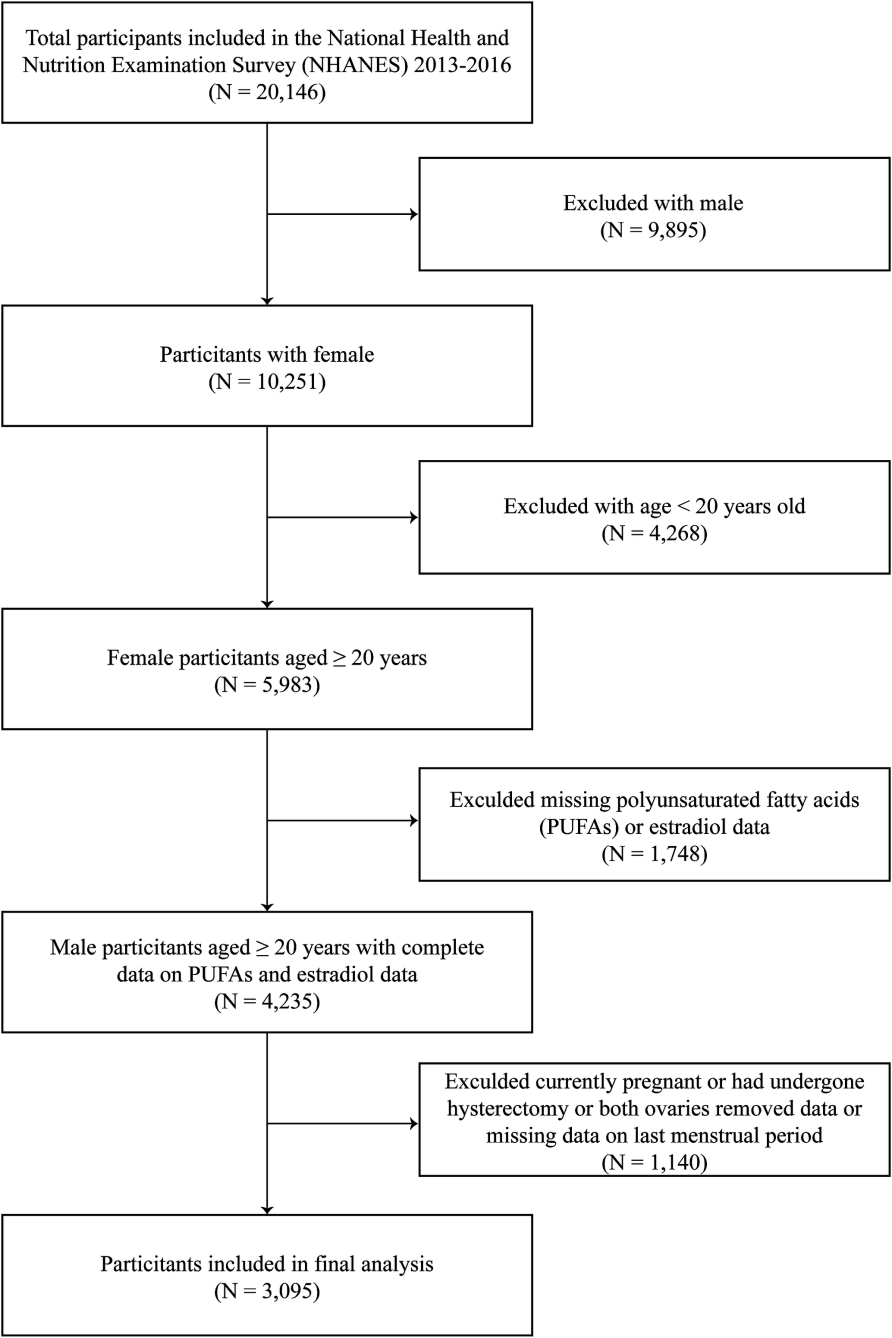
Study flowchart.

A total of 20,146 participants were included in this study. We excluded men (*n* = 9,895), participants aged <20 years (*n* = 4,268), and individuals with incomplete data on serum estradiol (*n* = 714) or PUFAs (*n* = 1,034). Additionally, those who were currently pregnant or had undergone hysterectomy or both ovaries removed or were breastfeeding (*n* = 924) and those with missing data on last menstrual period (*n* = 216) were also excluded. Overall, 3,095 participants with complete data were included in the final analysis (Figure 1).

### 2.2 Independent variable: PUFAs intake

Dietary data for NHANES were collected using 24-h dietary recall interviews conducted on two non-consecutive days, spaced 3 to 10 days apart (including weekends). These interviews provided estimates for energy, nutrients, and other food components. For this study, the intake of total polyunsaturated fatty acids (TPFAs), as well as omega-3 and omega-6 fatty acids, was calculated. Each PUFA intake was treated as a continuous variable for analysis.

### 2.3 Dependent variable: serum estradiol

Blood samples were drawn from participants after an overnight fast. The serum samples were processed, stored, and sent to the CDC’s National Center for Environmental Health, Division of Laboratory Sciences, in Atlanta, Georgia. Serum estradiol levels were measured using isotope dilution liquid chromatography–tandem mass spectrometry (ID-LC–MS/MS) (14).

### 2.4 Statistical analysis

The multivariable models accounted for potential confounders based on prior research (15). Covariates included age, race, educational level, ratio of family income to poverty (RIP), body mass index (BMI), smoking status, alcohol intake, hypertension, diabetes, high cholesterol, energy intake, and work activity status. The Department of Health and Human Services (HHS) poverty guidelines were used to calculate RIP. These guidelines are updated annually in the Federal Register. RIP was calculated by dividing family or individual income by the survey year’s specific poverty guidelines. BMI was categorized into three groups: <25 kg/m^2^, 25–29.9 kg/m^2^, and ≥30 kg/m^2^. Smoking status was determined by asking participants if they had smoked more than 100 cigarettes in their lifetime and if they currently smoked. Alcohol intake was assessed by asking whether participants had consumed at least 12 alcoholic drinks in the past year. Hypertension was diagnosed based on a physician’s diagnosis, use of antihypertensive medication, or measured systolic blood pressure ≥ 140 mmHg or diastolic blood pressure ≥ 90 mmHg. Diabetes was diagnosed either by a reported physician diagnosis or elevated fasting serum glucose levels. High cholesterol was defined as a physician diagnosis, use of cholesterol-lowering medication, or a total cholesterol level above 240 mg/dL. Menopausal status was determined by asking participants, “Has {you/your SP} had at least one menstrual period in the last 12 months? (Not including bleeding due to illness, hormone therapy, or surgery)” Women who answered “yes” were classified as non-menopausal. Among those who answered “no,” individuals who were pregnant, breastfeeding, or had undergone a hysterectomy or removal of both ovaries were excluded. The remaining women were classified as menopausal. Physical activity was categorized as vigorous or moderate based on whether the activity caused a significant or slight increase in heart rate or respiration for at least 10 min during the past week at work.

Participants were categorized according to the quartiles of estradiol. Continuous variables were presented as weighted means and standard errors, and categorical variables were presented as numbers and weighted percentages. Population characteristics of different estradiol quartiles were compared using linear regression for continuous variables and logistic regression for categorical variables. The association between PUFA intake and estradiol levels was analyzed using multivariable linear regression models. Subgroup analyses were conducted to explore stratified correlations between EPA and DPA intake and estradiol levels. The potential non-linearity between EPA and DPA intake and estradiol levels was assessed using smoothed curve fitting. A threshold effects analysis identified the inflection point in the non-linear relationship between EPA and DPA intake and estradiol levels, leading to the development of a two-piece regression model at this point. Intakes of all PUFAs and estradiol levels exceeding the mean by more than three standard deviations were classified as outliers. Missing data for covariates and outliers for PUFAs and estradiol were excluded from the statistical analysis. Because food intake differs between weekdays and weekends and using weights may disproportionately reflect weekend consumption, we used weights to minimize potential bias. All statistical analyses were performed using R^1^ and EmpowerStats.^2^ A *p*-value of less than 0.05 was considered statistically significant.

## 3 Results

### 3.1 Population characteristics

A total of 3,095 women aged 20 years or older with complete data were included in this study. Table 1 presents the weighted characteristics of participants across estradiol level quartiles. Participants had a mean age of 45.32 ± 16.50 years and a mean estradiol level of 59.60 ± 78.71 pg./mL. Lower estradiol levels were observed in participants who were older, had higher incomes, were Non-Hispanic White, had lower education levels, had a BMI below 25, consumed alcohol, were physically inactive, menopausal, and had low energy intake. Estradiol levels were also lower in participants who were without hypertension, diabetes, high blood cholesterol, or non-smokers. Additionally, significant differences in AA, EPA, and DPA distribution were also observed across estradiol quartiles.

**Table 1:** Baseline characteristics of the participants according to the estradiol quartile, weighted.

Characteristic	Q1 *N* = 771	Q2 *N* = 769	Q3 *N* = 775	Q4 *N* = 772	*p-*value
Age, years	58.51 ± 15.52	53.14 ± 15.54	35.23 ± 10.07	35.56 ± 9.04	<0.0001
PIR	3.16 ± 1.64	2.93 ± 1.63	2.69 ± 1.70	2.72 ± 1.64	<0.0001
Race, *n* (%)					<0.0001
Mexican American	51 (6.66)	59 (7.66)	94 (12.19)	111 (7.66)	
Other Hispanic	44 (5.68)	34 (4.43)	61 (7.87)	50 (4.43)	
Non-Hispanic White	570 (73.99)	531 (69.05)	429 (55.79)	421 (69.05)	
Non-Hispanic Black	46 (6.01)	84 (10.97)	101 (13.13)	109 (10.97)	
Other Race	59 (7.66)	61 (7.89)	85 (11.03)	78 (7.89)	
Education level, *n* (%)					<0.0001
Less than high school	123 (15.89)	92 (11.91)	81 (10.55)	96 (11.91)	
High school	135 (17.52)	215 (27.98)	122 (15.88)	150 (27.98)	
More than high school	513 (66.59)	462 (60.11)	566 (73.57)	523 (60.11)	
BMI, kg/m^2^					<0.0001
<25	356 (46.16)	143 (18.64)	230 (29.94)	299 (18.64)	
25–29.99	244 (31.65)	177 (23.07)	182 (23.69)	225 (23.07)	
≥30	171 (22.19)	448 (58.29)	357 (46.37)	245 (58.29)	
Hypertension, *n* (%)					<0.0001
Yes	371 (48.18)	339 (44.1)	138 (17.96)	144 (44.1)	
No	400 (51.82)	430 (55.9)	631 (82.04)	625 (55.9)	
Diabetes, *n* (%)					<0.0001
Yes	105 (13.65)	125 (16.22)	43 (5.55)	37 (16.22)	
No	666 (86.35)	644 (83.78)	726 (94.45)	732 (83.78)	
High cholesterol, *n* (%)					<0.0001
Yes	352 (45.66)	307 (39.98)	130 (16.87)	138 (39.98)	
No	419 (54.34)	462 (60.02)	639 (83.13)	631 (60.02)	
Smoking status, *n* (%)					0.0002
Yes	262 (33.99)	308 (39.99)	228 (29.66)	274 (39.99)	
No	509 (66.01)	461 (60.01)	541 (70.34)	495 (60.01)	
Alcohol intake, *n* (%)					<0.0001
Yes	502 (65.1)	500 (65.05)	550 (71.56)	573 (65.05)	
No	269 (34.9)	269 (34.95)	219 (28.44)	196 (34.95)	
Work activity status, *n* (%)					0.0073
Moderate	183 (23.75)	206 (26.8)	223 (28.95)	189 (26.8)	
Vigorous	94 (12.18)	98 (12.78)	127 (16.47)	112 (12.78)	
No	494 (64.08)	465 (60.42)	420 (54.58)	468 (60.42)	
Menopausal state, *n* (%)					<0.0001
Yes	622 (80.7)	537 (69.77)	89 (11.52)	51 (69.77)	
No	149 (19.3)	232 (30.23)	686 (88.48)	721 (30.23)	
Energy intake, kcal	1768.79 ± 652.56	1792.43 ± 658.39	1907.28 ± 683.17	1887.83 ± 717.58	<0.0001
LA	14.55 ± 6.86	14.40 ± 6.66	14.96 ± 6.97	14.79 ± 6.83	0.3737
ALA	1.54 ± 0.77	1.54 ± 0.79	1.54 ± 0.76	1.57 ± 0.76	0.8087
SDA	0.01 ± 0.01	0.01 ± 0.02	0.01 ± 0.02	0.01 ± 0.01	0.1175
AA	0.12 ± 0.07	0.13 ± 0.07	0.13 ± 0.08	0.13 ± 0.08	0.0248
EPA	0.02 ± 0.03	0.02 ± 0.04	0.02 ± 0.04	0.02 ± 0.04	0.0364
DPA	0.02 ± 0.01	0.02 ± 0.01	0.02 ± 0.01	0.02 ± 0.01	0.0014
DHA	0.05 ± 0.06	0.04 ± 0.07	0.05 ± 0.07	0.05 ± 0.08	0.0565
ω-3 PUFA	1.66 ± 0.82	1.67 ± 0.89	1.66 ± 0.82	1.69 ± 0.82	0.8298
ω-6 PUFA	14.67 ± 6.89	14.53 ± 6.68	15.10 ± 7.01	14.92 ± 6.85	0.3635
PFAT	16.49 ± 7.66	16.39 ± 7.55	16.91 ± 7.73	16.76 ± 7.55	0.5204

Continuous variables were presented as weighted means and standard errors, and categorical variables were presented as numbers and weighted percentages. Q1–Q4, quartile 1-quartile 4; PIR, ratio of family income to poverty; BMI, body mass index; AA, arachidonic acid; ALA, α-linolenic acid; DHA, docosahexaenoic acid; LA, linoleic acid; EPA, eicosapentaenoic acid; DPA, docosapentaenoic acid; SDA, octadecatetraenoic acid; TPFA, total polyunsaturated fatty acids.

### 3.2 Associations between AA, EPA, and DPA intake with estradiol levels

**Figure 2:**
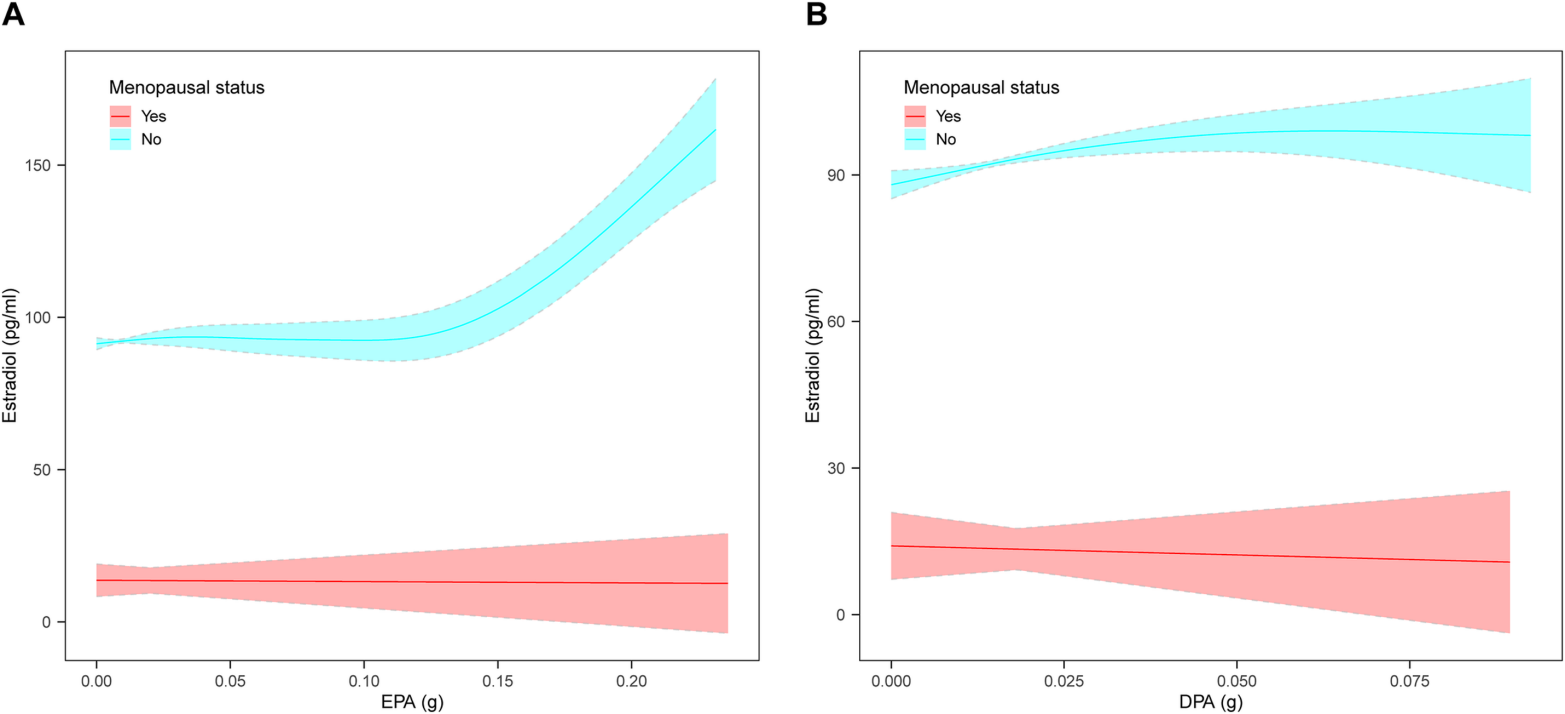
The non-linear relationship between EPA and DPA intake with estradiol levels stratified by menopausal status, weighted. **(A)** Non-linear relationship between EPA intake and estradiol levels. **(B)** Non-linear relationship between DPA intake and estradiol levels. The solid lines represents a smooth curve fit between the variables. The shaded area represents the 95% confidence interval of the fit. Age, race, education level, PIR, BMI, high cholesterol, diabetes, hypertension, smoking status, alcohol intake, energy intake, and work activity status were adjusted.

Weighted multivariate linear regression was applied to examine the relationship between AA, EPA, and DPA intake and estradiol levels, stratified by menopausal status (Table 2). Model 1 was adjusted for age, race, education level, PIR, and BMI. Model 2 included additional adjustments for high cholesterol, diabetes, hypertension, smoking status, alcohol intake, energy intake, and work activity status. AA intake was not significantly associated with estradiol levels. EPA intake was positively associated with estradiol levels in the crude model, model 1, and model 2, and this relationship was found in the menopausal (*β* = 78.08, 95% CI: 33.58, 122.58; *p* = 0.0006), non-menopausal (*β* = 287.61, 95% CI: 177.29, 397.94; *p* < 0.0001), and total-participant groups (*β* = 208.38, 95% CI: 139.81, 276.95; *p* < 0.0001). Positive associations with estradiol levels were also found in EPA intake in both the non-menopausal (*β* = 318.87, 95% CI: 28.93, 608.82; *p* = 0.0313) and total-participant groups (*β* = 208.03, 95% CI: 22.89, 393.18; *p* = 0.0277), remain significant after adjusting for partial or fully adjusted variables. Weighted smoothed curve-fitting analyses by menopausal status showed a non-linear relationship between EPA and DPA intake with estradiol levels after adjusting for all variables (Figure 2). In the two-piece regression model, EPA intake was positively associated with estradiol levels in non-menopausal women when EPA intake exceeded 0.09 (*β* = 938.26, 95% CI: 656.15–1220.37; *p* < 0.0001), and DPA intake showed a positive association in non-menopausal women when below 0.05 (*β* = 634.34, 95% CI: 211.32–1057.35; *p* = 0.0033) (Table 3).

**Table 2:** Multivariate linear regression revealed associations between AA, EPA, and DPA intake with estradiol levels stratified by menopausal status, weighted.

Exposure	Menopausal state				Total	
	Yes		No			
	*β* (95% CI)	*P*-value	*β* (95% CI)	*P*-value	*β* (95% CI)	*P*-value
AA						
Crude model	1.94 (−20.16, 24.04)	0.8635	−6.59 (−58.60, 45.42)	0.8038	−3.62 (−36.57, 29.33)	0.8295
Model 1	−9.83 (−31.31, 11.66)	0.3701	−2.13 (−55.54, 51.28)	0.9377	−4.48 (−38.72, 29.76)	0.7978
Model 2	−20.83 (−43.22, 1.57)	0.0686	3.28 (−52.91, 59.48)	0.9089	−7.49 (−43.46, 28.49)	0.6833
EPA						
Crude model	60.12 (14.35, 105.89)	0.0101	360.35 (256.24, 464.46)	<0.0001	245.48 (180.58, 310.37)	<0.0001
Model 1	82.86 (38.70, 127.03)	0.0002	283.25 (175.14, 391.37)	<0.0001	206.28 (138.77, 273.80)	<0.0001
Model 2	78.08 (33.58, 122.58)	0.0006	287.61 (177.29, 397.94)	<0.0001	208.38 (139.81, 276.95)	<0.0001
DPA						
Crude model	27.28 (−89.05, 143.61)	0.6459	296.01 (28.78, 563.24)	0.0301	203.90 (33.56, 374.24)	0.0190
Model 1	−28.97 (−142.34, 84.41)	0.6166	335.13 (56.45, 613.82)	0.0185	231.33 (52.72, 409.95)	0.0112
Model 2	−66.20 (−182.32, 49.92)	0.2641	318.87 (28.93, 608.82)	0.0313	208.03 (22.89, 393.18)	0.0277

Crude model: no covariates were adjusted.

Model 1: adjusted for age, race, education level, PIR, and BMI.

Model 2: adjusted for age, race, education level, PIR, BMI, high cholesterol, diabetes, hypertension, smoking status, alcohol intake, energy intake, and work activity status.

**Table 3:** Two-piecewise regression models of the relationship between EPA and DPA intake with estradiol levels, weighted.

Tow-piecewise linear model	Menopausal state			
	Yes		No	
	*β* (95% CI)	*P*-value	*β* (95% CI)	*P-*value
EPA				
Inflection point (K)	0.01		0.09	
EPA < K	223.07 (−328.19, 774.32)	0.1281	−201.88 (−426.05, 22.28)	0.0777
EPA > K	72.37 (22.88, 121.87)	0.0042	938.26 (656.15, 1220.37)	<0.0001
Log likelihood ratio		0.602		<0.001
DPA				
Inflection point (K)	0.02		0.05	
DPA < K	104.50 (−184.64, 393.64)	0.4788	634.34 (211.32, 1057.35)	0.0033
DPA > K	−154.90 (−334.93, 25.13)	0.0920	−731.87 (−1798.76, 335.02)	0.1790
Log likelihood ratio	0.202		0.044	

Age, race, education level, PIR, BMI, high cholesterol, diabetes, hypertension, smoking status, alcohol intake, energy intake, and work activity status were adjusted.

### 3.3 Subgroup analysis

**Figure 3:**
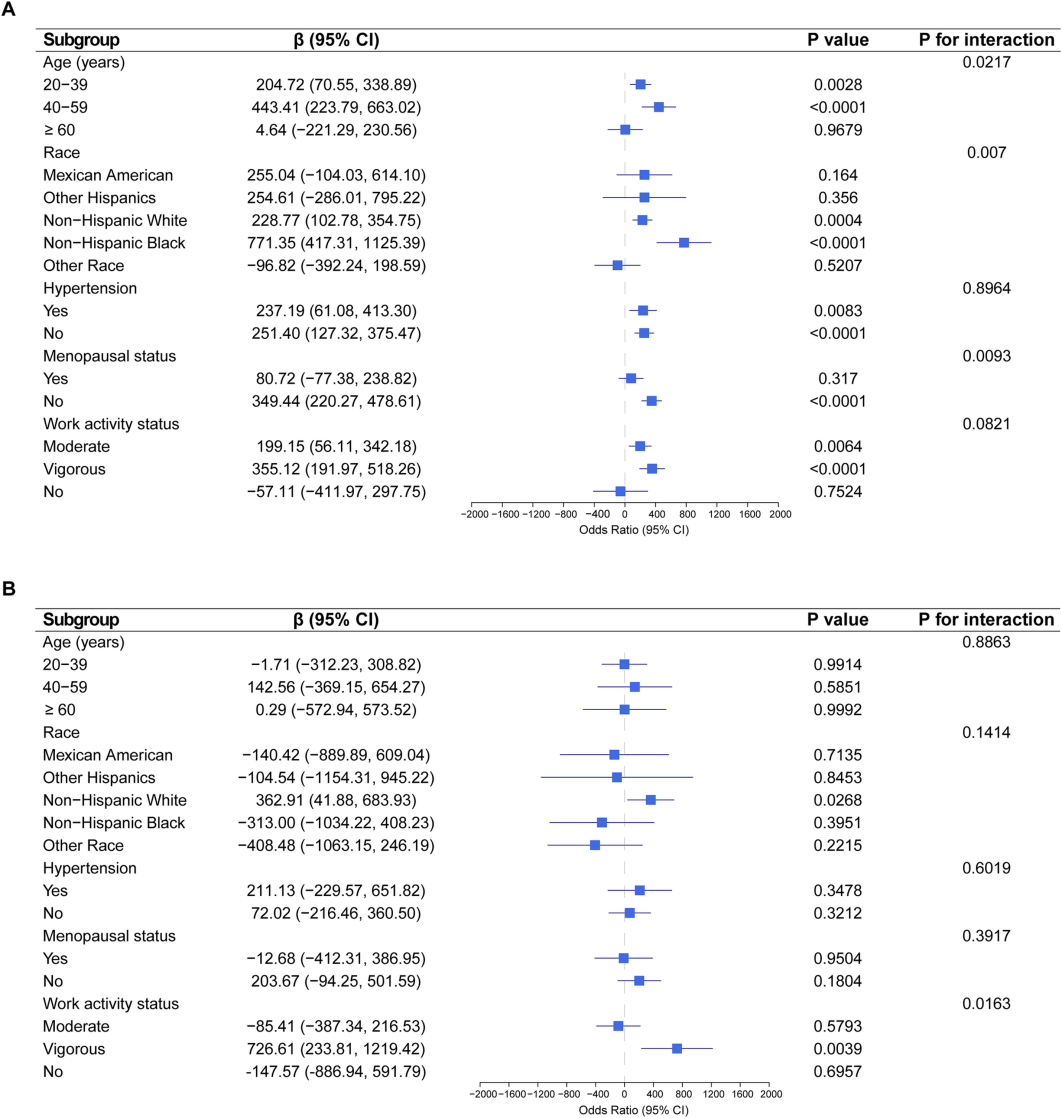
Association between EPA and DPA intake with estradiol levels severity, weighted. **(A)** Subgroup analysis of the association between EPA intake and estradiol levels. **(B)** Subgroup analysis of the association between DPA intake and estradiol levels. Each stratification was adjusted for age, race, education level, PIR, BMI, high cholesterol, diabetes, hypertension, smoking status, alcohol intake, energy intake, menopausal status, and work activity status, except the stratification factor itself.

Subgroup analysis indicated significant associations between EPA intake and estradiol levels in participants aged 20–39 years (*p* = 0.0028) and 40–59 years (*p* < 0.0001), Non-Hispanic White (*p* = 0.0004) and Non-Hispanic Black (*p* < 0.0001), and those in non-menopausal (*p* < 0.0001), moderate, or vigorous activity groups (Figure 3A). Additionally, significant associations were observed between DPA intake and estradiol levels in Non-Hispanic White individuals (*p* = 0.0268) and vigorous activity groups (*p* = 0.0039) (Figure 3B).

## 4 Discussion

In this cross-sectional study, we explored the association between PUFA intake and estradiol levels in a nationally representative sample of U.S. women. Our findings suggest that higher EPA and DPA intake in non-menopausal women is associated with elevated estradiol levels.

While an interaction between PUFA intake and estradiol levels is observed, the exact mechanisms remain unclear (16). Studies suggest that PUFAs are critical for maintaining cell membrane integrity, regulating inflammation, supporting gene expression, and influencing endocrine metabolism. PUFAs enhance cell membrane fluidity, potentially increasing the sensitivity and activity of estrogen receptors like ERα and ERβ, thus improving cellular responses to estradiol and hormone receptor signaling (17). PUFAs also modulate inflammation by influencing cells from the membrane to the nucleus (18). Omega-3 PUFAs, such as EPA and DHA, reduce the production of AA-derived eicosanoids, which are pro-inflammatory mediators. They incorporate into cell membranes, generating anti-inflammatory molecules such as resolvins and protectins. These effects may enhance ovarian function and promote estradiol synthesis and secretion (19). Conversely, omega-6 PUFAs like AA are precursors to pro-inflammatory molecules, such as prostaglandins and leukotrienes, which are critical in regulating inflammation (20).

Several studies have shown that estrogen levels in postmenopausal women are related to fatty acid levels. Stark et al. (21) found higher levels of C16:0, C16:1, and C20:3n-6 in postmenopausal women on hormone replacement therapy (MHT) compared to both premenopausal women and those not on MHT. Conversely, C22:0, C24:0, and C24:1 levels were lower in women on MHT. Sumino et al. (22) reported a significant increase in plasma DHA and EPA concentrations after 12 months of MHT in postmenopausal women. Piperi et al. (23) also observed significant changes in oleic acid and LA levels, along with reduced AA, in postmenopausal women on conjugated estrogens. Cybulska et al. (24) found higher C14:0 and C16:0 concentrations and lower C18:1n-9 and C20:4 levels in women on MHT compared to those not on therapy. These studies highlight that hormone replacement therapy significantly changes fatty acid composition in postmenopausal women, indicating a complex relationship between estrogen metabolism and fatty acid levels.

Different dietary patterns can affect estrogen levels in women in various ways. Bagga et al. (25) found that a low-fat, high-fiber diet decreased estradiol and estrone levels in healthy premenopausal women. Woods et al. (26) reported that a higher polyunsaturated to saturated fat ratio significantly reduced serum estrone sulfate levels. Young et al. (27) observed increased urinary estrone excretion in women on a low-fat, high omega-3 diet compared to a high-fat diet. In a study by Goldin et al. (28), vegetarians consumed less total fat and more dietary fiber than omnivores, and the results showed increased fecal excretion of estrogen and lower plasma concentrations of estrogen in vegetarians. Different dietary patterns, particularly fat (including unsaturated fats) and fiber intake, significantly impact estrogen levels in premenopausal women, with low-fat, high-fiber diets typically linked to lower estrone and estradiol concentrations.

Diet-influenced estrogen metabolism and excretion patterns may impact breast cancer risk (29). Elevated estrogen levels, high-fat diets, and decreased estrogen excretion are identified as key breast cancer risk factors (30). Elevated estrogen, high-fat intake, and reduced estrogen excretion are major breast cancer risk factors (31). Hormonal imbalances and elevated androgen levels caused by insulin resistance (IR) are considered primary drivers of polycystic ovary syndrome (PCOS) (32). Omega-3 supplementation improves IR, enhances lipid metabolism, lowers coronary heart disease risk, and improves reproductive outcomes (33, 34). Hohos et al. (35) showed a positive correlation between serum DHA, DPA, EPA levels, and primordial follicle count in mice, with FAT-1 mice on a high-fat diet having higher pregnancy rates and shorter gestation periods than wild-type mice. Wang et al. (36) found a reduced infertility risk with higher DHA intake. Elevated estrogen, high-fat intake, and insulin resistance-related hormonal imbalances significantly contribute to breast cancer risk and conditions like PCOS, whereas omega-3s can improve reproductive outcomes and reduce infertility risk.

Maintaining optimal estradiol levels through appropriate intake of EPA and DPA is essential to reduce inflammation, improve insulin resistance, promote lipid metabolism, reduce the risk of coronary heart disease, decrease the incidence of estrogen-related cancers such as breast and endometrial cancer, and improve reproductive outcomes (37–39). These findings provide strong evidence and valuable insights for developing dietary strategies that support women’s hormonal health. The use of weights in this study corrects for dietary differences on weekends and weekdays, ensuring that the results are representative of women across the United States. However, this study had several limitations. First, being cross-sectional, it could not establish causal relationships. Second, the study sample was limited to Americans. Third, the mechanisms by which these dietary intakes influence estradiol levels remain unclear, and further experimental studies are required.

## 5 Conclusion

This study showed that estradiol levels in non-menopausal women increased with higher dietary intake of EPA and DPA. This finding suggests that dietary composition significantly influences women’s reproductive health and hormone regulation. However, further research is needed to clarify the mechanisms underlying these associations.

## Data Availability

Publicly available datasets were analyzed in this study. This data can be found: NHANES 2013–2014 data: https://wwwn.cdc.gov/nchs/nhanes/ContinuousNhanes/Default.aspx?BeginYear=2013, NHANES 2015–2016 data: https://wwwn.cdc.gov/nchs/nhanes/ContinuousNhanes/Default.aspx?BeginYear=2015.

## Footnotes

^1^ https://www.R-project.org

^2^ https://www.empowerstats.net
